# *Burkholderia pseudomallei* Adaptation for Survival in Stressful Conditions

**DOI:** 10.1155/2018/3039106

**Published:** 2018-05-27

**Authors:** Taksaon Duangurai, Nitaya Indrawattana, Pornpan Pumirat

**Affiliations:** ^1^Department of Microbiology and Immunology, Faculty of Tropical Medicine, Mahidol University, Bangkok 10400, Thailand; ^2^Department of Companion Animal Clinical Sciences, Faculty of Veterinary Medicine, Kasetsart University, Bangkok 10900, Thailand

## Abstract

*Burkholderia pseudomallei* is a Gram-negative bacterium that causes melioidosis, which can be fatal in humans. Melioidosis is prevalent in the tropical regions of Southeast Asia and Northern Australia. Ecological data have shown that this bacterium can survive as a free-living organism in environmental niches, such as soil and water, as well as a parasite living in host organisms, such as ameba, plants, fungi, and animals. This review provides an overview of the survival and adaptation of* B. pseudomallei* to stressful conditions induced by hostile environmental factors, such as salinity, oxidation, and iron levels. The adaptation of* B. pseudomallei* in host cells is also reviewed. The adaptive survival mechanisms of this pathogen mainly involve modulation of gene and protein expression, which could cause alterations in the bacteria's cell membrane, metabolism, and virulence. Understanding the adaptations of this organism to environmental factors provides important insights into the survival and pathogenesis of* B. pseudomallei*, which may lead to the development of novel strategies for the control, prevention, and treatment of melioidosis in the future.

## 1. Introduction

*Burkholderia pseudomallei* is a Gram-negative bacterium that is the causative agent of melioidosis, an infectious disease of public-health significance in Southeast Asia and Northern Australia [1, 2]. In an endemic area, a mortality rate of 40% among septicemic melioidosis patients has been reported in Thailand [3]. In Australia, the mortality rate was about 21% [3]. In nature,* B. pseudomallei* can survive in diverse environmental niches, indicating an ability to sense and respond to changes in the environment via specific survival mechanisms. This review provides information on the adaptations of* B. pseudomallei* in response to various hostile environmental stress factors, such as salinity, oxidation, and iron content, as well as its adaptation in target cells. Essential changes in gene and protein expression to enable* B. pseudomallei* to adapt are explored. Future directions for* B. pseudomallei* research are also discussed.

## 2. Background Information for* B. pseudomallei*

*B. pseudomallei* was discovered in 1911 by Whitmore and his team as a bacterial agent associated with “glanders-like” disease [2]. Many years later, this bacterium was proven to cause melioidosis [4]. This life-threatening disease presents with a wide range of nonspecific signs and symptoms, including fever, pneumonia, acute septicemia, and chronic localized infection [4, 5]. Chronic infection can cause abscesses in various internal organs, such as the lungs, liver, spleen, kidneys, prostate gland, and skeletal muscles [5]. The routes of transmission of* B. pseudomallei* include inoculation via skin abrasion, inhalation, and ingestion [6]. Patients with diabetes, thalassemia, or renal disease or people who work in paddy fields have been reported to have a higher risk of melioidosis [7]. However, healthy individuals with no obvious risk factors can also become infected, albeit with significantly lower risk. Without appropriate treatment, the septicemic form of melioidosis can develop and is associated with >90% mortality rate [2]. Currently, no effective vaccine exists to prevent melioidosis.

*B. pseudomallei* is found in a wide range of ecological niches, including soil and surface water, and has also been found to adhere to the roots of legumes [8]. The wide variety of* B. pseudomallei* habitats may help explain the persistence of this bacterium in endemic areas. Several studies have indicated that climatic, physical, chemical, and biological factors could control the proliferation and survival of* B. pseudomallei* in the environment. For example, many studies have demonstrated an association between the frequency of recorded melioidosis cases and rainfall-related events [9–11]. Merritt and Inglis suggested that the levels of cloud cover during rainfall correspond to the levels of soil moisture and might provide appropriate conditions for the survival of* B. pseudomallei* [12]. Dense cloud cover may provide* B. pseudomallei* with protection from bactericidal UV wavelengths in sunlight [12]. Soil is considered a major reservoir of* B. pseudomallei,* a saprophytic organism. Thus, the physiochemistry of the soil is likely a key factor supporting the survival of* B. pseudomallei*. Manivanh et al. [13] found the prevalence of* B. pseudomallei* to be high at soil depths > 30 cm with high water content and low total nitrogen, carbon, and organic matter. Tong et al. [14] showed that* B. pseudomallei* can survive in soils with 20% moisture for 439 days, which is longer than without water, where bacterial survival was only 30 days. This may be because soil moisture supports the availability of bacterial nutrients and membrane integrity [8]. It has been demonstrated that* B. pseudomallei* can persist in harsh-environment, nutrient-limited settings, such as low-iron environments [15]. Conversely, Musa et al. [16] found that soil containing high levels of iron was strongly associated with* B. pseudomallei* persistence. It is possible that iron can regulate the expression of respiratory enzymes in the biological processes involved in* B. pseudomallei* survival [17]. Biological factors are important for* B. pseudomallei *to persist in the environment. It is evident that free-living amebae are an environmental reservoir for* B. pseudomallei.* The important amebae for* B. pseudomallei* are* Acanthamoeba *spp.,* Hartmannella *spp., and* Naegleria *spp. [18]. This seems to be advantageous for* B. pseudomallei* survival in harmful conditions.* B. pseudomallei* has reportedly survived inside amebae in an environment contaminated with disinfectants and antibiotics [19]. However, not all ameba species can facilitate the persistence and dispersal of a particular bacterial pathogen in the environment, since some amebae isolated from endemic areas can antagonize* B. pseudomallei* [20], including* Paravahlkampfia ustiana*,* Acanthamoeba* spp., and isolate A-ST39-E1.

*B*.* pseudomallei *is likely to be constantly exposed to a variety of stressful conditions, forcing it to adapt and to survive in environmental niches.* B. pseudomallei* contains a number of genes that are important for survival and adaptation. The genome sequence of* B. pseudomallei* has revealed that the bacterium has two large chromosomes (4.07 and 3.17 Mb), containing at least 16 genomic islands [21]. The major chromosome carries many genes associated with core functions, such as cell growth and metabolism, whereas the smaller chromosome carries genes encoding accessory functions, such as those required for bacterial adaptation and virulence. In addition to survival in the environment, upon infection,* B. pseudomallei* has the ability to survive inside a variety of host cells, and this is mediated by several virulence factors, such as type 3 secretion system (T3SS), type 5 secretion system (T5SS), type 6 secretion system (T6SS), lipopolysaccharide (LPS), and flagella, as well as numerous bacterial products and enzymes [22, 23]. These factors contribute to* B. pseudomallei* pathogenesis [22] and also occasionally facilitate its adaptation under hostile environmental conditions. This issue will be discussed further below.

## 3. Molecular Mechanisms for* B. pseudomallei *Adaptation in the Presence of Hostile and Environmental Factors

The unusual ability of* B. pseudomallei* to survive for months or years in the environment is evident by the persistence of this bacterium in melioidosis-endemic areas [8]. Indeed,* B. pseudomallei* showed the ability to survive in adverse environments, including lack of nutrients [24], limited oxygen [25, 26], and exposure to high salt concentrations [27, 28] and oxidative agents [29, 30]. Importantly,* B. pseudomallei* is able to sequester within human macrophages and lymphoreticular organs in a dormant or quiescent state for many years [8]. Various environmental and hostile stresses are critical factors contributing to the adaptive survival mechanisms of* B. pseudomallei*. Like other bacteria,* B. pseudomallei* possesses various mechanisms to modulate its gene expression for survival under stress (Table 1). The adaptation of* B. pseudomallei *to stress includes modulation of the expression of genes encoding important proteins, such as short-chain dehydrogenase/oxidoreductase (SDO) [31], acyl-CoA dehydrogenase [27],* Burkholderia* secretion apparatus (Bsa) T3SS [27], beta-lactamase-like protein [28], sigma factor E (RpoE) [27, 32], and heat-shock proteins [27, 32] for salt stress; KatG and KatE catalase enzymes [33], sigma factor S (RpoS) [34], succinyl-CoA: 3-ketoacid CoA transferase (SCOT) [34], and DpsA [35] for oxidative stress; Fur [36], pyochelin [37], pyoverdine [37], ornibactin [37], cepabactin [37], and biofilm formation-associated regulator [38] for iron stress; and ATP synthases [25], polyhydroxybutyrate synthase [25], pyruvate dehydrogenase [25], acetate kinase [25], alcohol dehydrogenase [25], motility-mediated proteins [25], stress-related proteins [25], and virulence factors [25] for oxygen stress. Most of these stress-response proteins have been observed to react with sera from melioidosis patients [39], potentially indicating their important roles in the adaptation of bacteria to survive under ecologically stressful conditions.

### 3.1. Adaptation to Salt Stress

**Figure 1:**
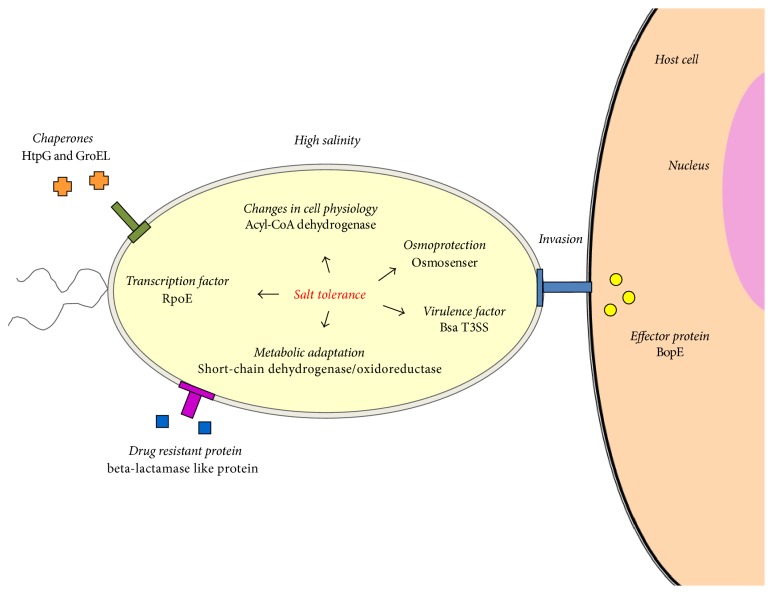
Mechanism of* Burkholderia pseudomallei *adaptation in response to high salt stress.

In Thailand, the highest incidence of melioidosis and the highest prevalence of* B. pseudomallei *are found in the northeast, where saline soil and water are abundant [15]. This raises the possibility that* B. pseudomallei* can adapt to saline conditions and gain a selective ecological advantage over other soil microorganisms. Consistent with this,* B. pseudomallei *infection has been reported in cystic fibrosis patients, who have higher salt concentrations in their lungs than healthy individuals [27]. Several studies have revealed that exposure to high salinity influences* B. pseudomallei* survival and virulence, by adjusting the expression of genes and proteins involved in bacterial physiology, virulence, and metabolism [27, 31, 40]. A possible mechanism by which* B. pseudomallei* adapts to counter salinity stress is shown in Figure 1.

In general, when bacteria encounter salt stress, they recognize environmental stress with an osmosensor [41]. It has been reported that an adenylate cyclase (CyaB) acts as an osmosensor in the Gram-negative saprophytic bacterium* Myxococcus xanthus* [42]. Under salt stress, the expression of* B. pseudomallei* adenylate cyclase is increased [27]. Adenylate cyclase might function as an osmosensor in* B. pseudomallei* or might be involved in the transmission of the signal. However, the exact role of adenylate cyclase in adaptation to salt stress is still unknown.

Under salt-stress conditions, there is evidence of severely impaired growth and morphology in* B. pseudomallei *[27, 43]. In our previous study,* B*.* pseudomallei* K96243 demonstrated growth impairment during culturing in LB containing 470 mM NaCl [27]. Moreover, morphological alteration from rod to coccoid was found in* B. pseudomallei* adaptation to high salt stress [43]. Changes from the rod to the coccoid form increase the cell membrane surface, which might benefit nutrient uptake by the bacterium [43].* B. pseudomallei* also changes its membrane in response to salt stress.* B. pseudomallei *showed upregulated expression of Acyl-CoA dehydrogenase during high salt stress [27]. Acyl-CoA dehydrogenases are involved in changes in bacterial membrane fluidity during salt tolerance [44]. Acyl-CoA dehydrogenases may therefore play a role in adjusting the bacterial membrane lipid composition, modifying the types of fatty acids present, and altering the structures of phospholipids when* B. pseudomallei* encounters high salt levels.

The influences of salt stress on the pathogenicity of* B. pseudomallei* have been studied intensively [27, 28]. NaCl-exposed* B. pseudomallei* secreted many effector proteins, including the beta-lactamase-like protein, which led to greater survival after treatment with beta-lactam antibiotics [28]. Indeed, high salt stress resulted in the increased invasion of* B. pseudomallei* into A549 human lung respiratory epithelial cells, by increasing the expression and secretion of Bsa T3SS proteins [27]. Bsa T3SS is an important virulence factor for* B. pseudomallei* invasion and intracellular replication. High salt stress can increase the transcription of* bipD* and* bopE* genes, which encode the Bsa translocon component and the virulence-associated effector involved in actin dynamics, respectively. Besides, the increased Bsa T3SS may participate in the enhanced plaque formation of* B*.* pseudomallei* observed after exposure to NaCl [31].

An alternative model of T3SS triggering under salt stress has been linked with MucA-mediated coordination of alginate production in* P. aeruginosa* [45]. Alginate production is known to be activated by high salt conditions [46]. A comparison of global gene expression of* mucA* mutant- and wild-type strains under T3SS-inducing conditions showed the downregulation of T3SS genes and upregulation of genes involved in alginate biosynthesis. Under high salt conditions, the upregulation of sigma factor* rpoE* was observed in* B. pseudomallei*, suggesting a role for* rpoE* in tolerance to environmental stress [30]. Similarly, the upregulation of* rpoE *was observed; it was postulated to be involved in the regulation of T3SS in* P. aeruginosa*. Therefore,* rpoE* might play a role in controlling* B. pseudomallei* T3SS expression under high-salinity conditions, as described for* P. aeruginosa*.

*B. pseudomallei* can also alter bacterial metabolism under salt stress by upregulating the expression of short-chain dehydrogenase/oxidoreductase (SDO) [31]. SDO, an important enzyme in the metabolic pathways [47], catalyzes the NADPH-dependent reduction of many compounds, such as sugars, aldehydes, and ketones [48]. Recently, the induction of SDO activity during salt stress has been shown to be linked to the adaptation and pathogenesis of* B. pseudomallei*, by facilitating the invasion of host cells [31]. However, further experiments are required to investigate the underlying mechanism.

More recently, salt stress was found to increase thermal resistance, oxidative resistance, and plaque formation, while decreasing the motility of* B. pseudomallei* [32]. The resistance of* B. pseudomallei* to heat and oxidative stress may result from the increased gene expression of stress-response cellular components, such as sigma factor* rpoE*, and heat-shock proteins* groEL* and* htpG* in* B. pseudomallei* under high-salinity conditions [32]. Inactivation of the* rpoE* operon increased the susceptibility of* B. pseudomallei *to killing by menadione and hydrogen peroxide (H_2_O_2_) and high osmolarity [30]. Furthermore, it has been demonstrated that* rpoE* regulated a heat-inducible promoter of the* rpoH* gene in* B. pseudomallei* [49]. These data imply that RpoE plays an important role in the increased resistance of* B*.* pseudomallei* in response to heat and oxidative stress. Taken together, the evidence suggests that adaptive changes induced by salt stress may aid* B. pseudomallei* survival and/or persistence in various environments.

### 3.2. Adaptation to Oxidative Stress

**Figure 2:**
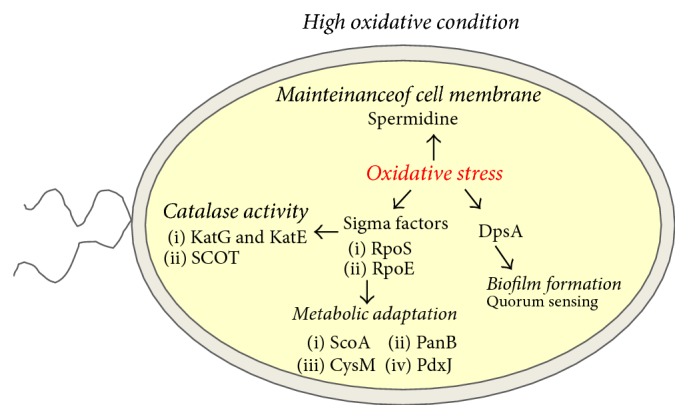
Mechanism of* Burkholderia pseudomallei *adaptation in response to oxidative stress.

Reactive oxygen species (ROS) can be generated by living organisms and chemical processes that occur in the environment. For example, H_2_O_2_ is produced by the oxidation of metals and sulfur species, or by UV radiation [50]. Some organic peroxides are produced by plant [51] and animal hosts [52] as defense mechanisms against microbial pathogens [53]. ROS play a role in controlling early* B. pseudomallei* infection by threatening and inhibiting the intracellular growth of* B. pseudomallei*. Thus, to survive,* B. pseudomallei* must possess a mechanism to adapt to this hostile factor, as shown in Figure 2.

The response of* B. pseudomallei* to oxidative stress is regulated by sigma (*σ*) factors [54], which are groups of proteins required for RNA synthesis. *σ* factors bind to the core of RNA polymerase to initiate RNA synthesis [55]. *σ* factors can be classified into 2 families: the *σ* 54 family and the *σ* 70 family [56]. Members of the *σ* 70 family are responsible for the expression of all essential genes, while members of the *σ* 54 family are mostly involved in nitrogen metabolism-associated genes.* B. pseudomallei* contains several *σ* factors, including RpoC (*σ*C) [57], RpoN (*σ*N) [58], RpoE (*σ*E) [30], and RpoS (*σ*S) [34]. RpoE and RpoS are members of the *σ* 70 family which play an important role in response to extracellular stress [55]. The* rpoE *gene of* B. pseudomallei* was activated during bacterial exposure to oxidative stress conditions [29]. When* B. pseudomallei* is exposed to H_2_O_2_-induced oxidative stress, the *σ*E regulon turns on the expression of the* speG* gene involved in maintaining the levels of the polyamine, spermidine [29]. Spermidine helps* B. pseudomallei* to survive oxidative stress and plays vital roles in cell survival, by synchronizing biological processes such as Ca^2+^, Na^+^, and K^+^ -ATPase, to maintain membrane potential and control intracellular pH and volume during oxidative stress [59]. In addition to* rpoE* activation, the* B. pseudomallei rpoS *gene was activated during bacterial exposure to oxidative stress conditions [54]. RpoS controls the expression of genes encoding KatG and KatE catalase enzymes when* B. pseudomallei* is exposed to H_2_O_2_ [33]. RpoS also upregulates proteins involved in the response to oxidative stress, including succinyl-CoA: 3-ketoacid-coenzyme A transferase subunit A (ScoA), cysteine synthase B (CysM), 3-methyl-2-oxobutanoate hydroxymethyltransferase (PanB), and pyridoxal phosphate biosynthetic protein (PdxJ) and other proteins, which are universal-stress- and hypothetical oxidative-stress-responsive proteins [34]. When* B. pseudomallei *is exposed to oxidative stress, RpoS downregulates SCOT (a dimeric enzyme containing subunits A and B) expression to reduce endogenous ROS [34]. This mechanism enables the bacterium to reduce ROS intracellularly.

In addition to the genes and proteins regulated by *σ* factors mentioned above, the DNA-binding protein DpsA is involved in* B. pseudomallei* adaptation during exposure to oxidative stress [35]. DpsA plays a major role in protecting* B. pseudomallei* from oxidative stress through increased transcription of the* katG *(catalase peroxidase) promoter [60]. Moreover,* dpsA* gene expression is regulated in a cell population density-dependent manner via N-acylhomoserine lactone- (AHL-) dependent quorum sensing (QS). In several Gram-negative bacteria, QS is involved in biofilm formation, which is dependent on LuxI-type AHL synthases and LuxR-type transcriptional regulator proteins [35].* B*.* pseudomallei* can produce biofilm, which may offer protection against hostile conditions, such as antibiotic treatment, salinity, and immune response [7, 8, 61]. Although it remains to be determined how* B. pseudomallei* triggers QS systems after exposure to oxidative stress, it is likely that biofilm formation and virulence-factor production are important survival mechanisms for* B. pseudomallei* in response to oxidative stress [35, 60].

### 3.3. Adaptation to Iron Concentrations

**Figure 3:**
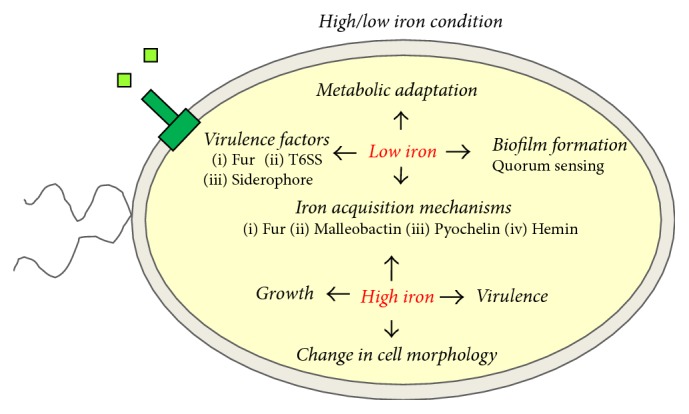
Mechanism of* Burkholderia pseudomallei *adaptation in response to iron content.

Iron is an essential microelement that contributes to the adaptation of* B. pseudomallei* to specific environmental niches, such as the soil and the host. The proposed adaptation of the* B. pseudomallei* response to iron content is shown in Figure 3. Iron plays a role as a cofactor of enzymes in cellular functions and metabolic processes. Therefore, an increase in iron concentration enhances the growth of* B. pseudomallei* [62], changes the bacterial morphology from rod form to coccoid form, and increases biofilm formation [61]. Furthermore,* B. pseudomallei* intracellular survival and MNGC formation cultured in A549 cell lines supplemented with iron are greater than in a non-iron-supplemented group [63]. The plaque-forming efficiency that indicates the severity of* B. pseudomallei* infected HeLa cells is increased in the presence of iron [63]. This raises the possibility that conditions with increased iron stores, such as thalassemia, are considered to increase the risk of acquiring melioidosis. In Thai adults, thalassemia was associated with an 11-fold increase in melioidosis compared with other patients with sepsis [64]. A recent study, reporting from the period from 2001 to 2010, showed that thalassemia was a major risk factor for melioidosis among Malaysian children [65]. Meanwhile, low-iron conditions were found to limit the growth of* B. pseudomallei* [63] and decrease the virulence of this bacterium [66]. A study of mice infected with* B. pseudomallei* showed that iron deprivation decreases bacterial load in visceral organs such as the lungs, liver, and spleen, which was associated with the improved survival of mice [66]. These studies indicate that iron is an important factor in* B. pseudomallei* infection.

Generally, free iron is limited in physiological habitats and sequestered by the host by the iron‐binding proteins such as transferrin and lactoferrin. As a result, the bacterium must employ mechanisms of iron uptake regulation for survival under iron-restricted conditions. The iron regulators function under low-iron conditions by the expression of genes encoding an iron-acquisition system. Among these are the iron regulator gene* “fur”* (ferric uptake regulator), genes coding for iron-binding proteins, that is, siderophore (also called malleobactin), pyochelin, pyoverdine, ornibactin, cepabactin, and heme-hemin receptors, as well as a variety of genes involved in the metabolic pathway, that is, ferredoxin, NADH dehydrogenase, cytochrome oxidase, and ATP synthases [17, 37, 67].

Loprasert and coworkers reported that* B. pseudomallei* adapts itself in iron-limited conditions by upregulating the iron-acquisition system via the* fur* gene, which encodes a regulatory protein, Fur (ferric uptake regulatory) protein [36]. The Fur protein represses the transcription of iron-regulated promoters in response to increased intracellular iron concentrations. The Fur protein is also involved in the expression of toxins and bacterial virulence determinants in other bacteria [67, 68]. In* B. pseudomallei*, the Fur protein functions as a positive regulator of FeSOD (ferric-superoxide dismutase) and peroxidase to reduce free radicals and oxidative stress [36]. These enzymes influence the virulence of many bacteria [69]. However, the role of Fur in the virulence of* B. pseudomallei* has not, to date, been demonstrated.

The primary siderophore (malleobactin) plays an important role in iron uptake and regulation in* B. pseudomallei*. In addition to malleobactin,* B. pseudomallei *also produces many secondary siderophores, such as pyochelin, pyoverdine, and ornibactin, to control iron uptake [70]. Siderophores have been shown to correlate with the increased virulence of* B. pseudomallei* [71]. However, the mechanisms of these siderophores are still unclear in* B. pseudomallei. *In closely related* B. cenocepacia*, it has been reported that iron uptake via secondary siderophore, ornibactin, depends on the* pvdA* gene, encoding ornithine N^5^-oxygenase, and the* orbA *gene, encoding the outer membrane receptor [72]. An* orbA* is involved in ferric-ornibactin complex transport. Moreover,* pvdA* and* orbA* genes are required for the virulence of* B. cenocepacia *[72, 73].

Moreover,* B. pseudomallei* heme uptake (Bhu/Hmu) system encoded by* BPSS0240–BPSS0244* genes was found to be upregulated during growth under low-iron conditions [17]. This system requires heme-hemin receptors that are present on the outer membrane of* B. pseudomallei*. In addition, the heme uptake system requires the action of the cytoplasmic membrane-anchored TonB-ExbB-ExbD complex to energize transport of these iron sources (ATP-binding cassette transporter systems) [70, 74]. The importance of the Bhu/Hmu system was investigated by Kvitko and coworkers [70], who showed that the deletion of the* bhu/hmu* locus affected the ability to utilize heme or hemoglobin as iron sources.

Under low-iron conditions,* B. pseudomallei* switched its metabolic pathways by obtaining energy from nitrogen metabolism and electron transport for survival [17]. It was found that* BPSS0495, *a gene encoding the nitroreductase enzyme responsible for nitrogen compound metabolism, was highly upregulated among* B. pseudomallei* grown in iron-restricted conditions.* B. pseudomallei* obtains energy from electron transport with the expression of bacterioferritin-associated ferredoxin genes under low-iron conditions.* B. pseudomallei* may use ferredoxin as an electron donor [17].

In addition to the iron-acquisition system,* B. pseudomallei* adapts its virulence-associated phenotypes during survival in low-iron conditions. One study reported that the biofilm formation-associated regulator* (bfmR)* gene was upregulated under low-iron conditions [38]. It is possible that* B. pseudomallei* adaptation might employ biofilm formation for survival [61]. This concurs with a previous study that found that the biofilm of* B. pseudomallei* increased bacterial adherence to host cells [75]. The T6SS genes, which encode proteins that facilitate cell-to-cell spreading, are reportedly induced by iron deprivation [76]. Taken together, it is reasonable to hypothesize that iron-acquisition mechanisms and T6SS might contribute to the control of* B. pseudomallei* adaptation after exposure to iron-limited conditions.

### 3.4. Adaptation in Host Cells

During the infection process,* B. pseudomallei* encounters various stress factors, such as nutrient restriction, oxygen limitations, and host defense mechanisms. Thus,* B. pseudomallei* must adapt itself to survive in the host using several mechanisms. Successful adaptation results in the survival of* B. pseudomallei* in a variety of phagocytic and nonphagocytic cells [77]. During* B. pseudomallei* survival in the host cells, several genes, including virulence factors, are functionally modulated [22, 78]. Several components of T3SS were found to be involved in many stages of* B. pseudomallei* pathogenesis, including invasion (BopB, BopC, BopE, BipB, BipC, BipD, and BsaZ), phagosome escape (BopC, BipC, BsaM, BsaQ, BsaU, and BsaZ), intracellular survival (BopA, BopB, BopC, BipC, BsaQ, and BsaZ) and cell–cell spreading (BipB, BipC, BsaS, BsaZ, and ChbP) [40, 79–89]. T6SS-1 was shown to modulate the intracellular growth of* B. pseudomallei* via the sensor regulators, BprC and VirA-VirG (VirAG) [90]. In addition to T3SS, T6SS plays a major role during bacterial transition from the phagosome to the cytosol [90]. Furthermore, the expression of* bimA* (*Burkholderia* intracellular motility A), which is translocated by the T5SS, was increased at 2 to 6 h after infection.* B. pseudomallei* BimA is required for intracellular actin-based motility and cell-to-cell spread [91]. In addition,* B. pseudomallei* modulates the bacterial surface structures to avoid host immune system recognition by downregulating genes involved in capsular polysaccharide biosynthesis, polysaccharide biosynthesis, LPS biosynthesis, flagella assembly, and chemotaxis during survival inside host cells [23].

*σ* factor genes were also found to be involved in* B. pseudomallei* survival in host cells. One of the* B. pseudomalleiσ* factor genes,* rpoS*, is reported to be a key regulator for intracellular survival under carbon starvation and oxidative stress [54]. In general, RpoS acts as a positive transcriptional regulator of* oxyR* and* dpsA* expression. Under oxidative stress,* rpoS* upregulated expression of* oxyR* and the* katG–dpsA* operon.

*B. pseudomallei* has various metabolic mechanisms to obtain the available host nutrients for its own proliferation. In a challenge study of oxygen-limited conditions, many genes of* B. pseudomallei* were induced. Among those were genes encoding proteins in arginine and pyruvate fermentation (*aceE*,* arcD*, and* tatA*), ATP synthases (*atpA *and* atpD*), electron transport proteins (*aarC*,* cydA*,* cydB*,* mocA*, and* BPSL1260*), flagella-mediated motility (*flgA*,* flgC*,* flgK*,* flgM*,* fliF*,* fliJ*,* fliK*, and* pilT*), stress-related proteins (*clpB*,* rpoH*, and* rpoS*), virulence factors (*bopE*,* bipC*,* bipD*,* orgA*, and* pilA*), and polyhydroxybutyrate synthase (*bdhA-2*). These findings suggest that* B. pseudomallei *presents an excellent transcriptional network that allows it to respond to conditions of limited oxygen [25]. Hypoxic conditions also lead to the repression of genes involved in ribosomal biogenesis, suggesting an overall reduction in protein synthesis during oxygen depletion, which is related to reduced bacterial growth rate [25].

In contrast to the challenge study, during the early stage of macrophage infection, a study has shown that genes involved in metabolism, glycolysis, and oxidative phosphorylation were downregulated while genes responsible for anaerobic metabolism, including pyruvate dehydrogenase, acetate kinase, and alcohol dehydrogenase, were induced [92]. This might be because the bacteria need to adjust their metabolism in response to the hypoxic conditions in the host cells [92]. Genes involved in benzoate degradation were also upregulated, suggesting that intracellular* B. pseudomallei* utilize aromatic compounds as a carbon source.

These findings demonstrate the importance of environmental or host conditions in the regulation of* B. pseudomallei *intracellular survival. However, the mechanism of regulation of gene expression requires further investigation.

## 4. Conclusions and Future Perspectives

This review outlines our current knowledge of the adaptive mechanisms that enable* B. pseudomallei* to survive and grow under various conditions, such as salinity, oxidative stress, altered iron concentrations, and host-associated conditions. Adaptations allow the organism to tolerate hostile environments and may also provide other advantages, such as increased bacterial virulence, evasion of host defenses, reduction in free radicals, and decreased growth rates for latent infections.

*B. pseudomallei* possesses several mechanisms by which it senses sources of stress in the environment and in the host, and then, depending on the type of stress, bacterial adaptation leads to the modulation of changes in the expression of the genes and proteins involved in metabolism, ion transport systems, and virulence factors. Increasing evidence strongly supports the adaptation of* B. pseudomallei *within the host, including pathways involved in environmental survival, which lead to bacterial persistence under adverse conditions. This insight is useful for understanding the underlying mechanisms that are important for the intracellular and extracellular adaptation of* B. pseudomallei*. This precise knowledge therefore opened the doors for novel targets for the treatment and prevention of melioidosis.

The potential sources of stress encountered by* B. pseudomallei* are not limited to those reviewed here. Further studies of* B. pseudomallei* adaptation under other stress conditions, such as acidity, osmotic stress, ammonia accumulation, antibacterial agent exposure, the presence of nitric oxide, and abscess condition, will also contribute to our understanding of bacterial survival and persistence. Other bacterial components that may be altered during* B. pseudomallei* adaptation following exposure to stress should also be investigated. The adaptation of* B. pseudomallei* to survival in ecological niches is a complex multifactorial process that depends on more than one environmental factor. However, currently, no reports show that* B. pseudomallei* can adapt in response to simultaneous exposure to multiple sources of stress. Such studies are needed to reflect actual environmental challenges and to provide a better understanding of* B. pseudomallei* survival and pathogenesis.

## Figures and Tables

**Table 1:** Stress-regulated genes for *Burkholderia pseudomallei *adaptation.

Type of stress	Stress regulated genes		
	Membrane structure	Metabolism	Virulence
*Salinity*			
High level	gene encoding Acyl-CoA dehydrogenase [27] and *cyaB* [27]	gene encoding SDO [31]	*bsa*-encoded genes (*bipD* and *bopE*) [27],* rpoE* [27], *mucB* [27], *groEL *[32], and *htpG *[32]
Low level	-	-	-
*Oxidative condition*			
High level	*rpoE *[29] and *speG* [29]	*rpoS* [34], *scoA *[34], *cysM* [34], *panB *[34], *pdxJ *[34], gene encoding SCOT [34], and genes encoding KatG and KatE [33]	*dpsA* [35]
Low level	-	-	-
*Iron *			
High level	-	*fur* [36]	*mba *[37],* pch *[37], and *bhu/hmu *[17]
Low level	-	*BPSS0495 *[17]	*fur* [36], *bfmR *[38], and genes encoding T6SS [76]
*Oxygen *			
High level	-	*-*	-
Low level	-	Genes encoding ATP synthases (*atpA* and *atpD*), arginine and pyruvate fermentation (*aceE*, *arcD *and* tatA*), electron transport proteins (*aarC,cydA, cydB, mocA,* and *BPSL1260*), and polyhydroxybutyrate synthase (*bdhA-2*) [25]	Genes encoding flagella-mediated motility (*flgA, flgC, flgK,flgM, fliF, fliJ*,* fliK,* and *pilT*), stress-related proteins *(clpB, rpoH*, and *rpoS*), and virulence factors (*bopE*,* bipC*,* bipD*, *orgA*,and *pilA*) [25]
